# Co-Creating in Close Collaboration with Community Stakeholders: Transferable Lessons Learned from the Development of a Group-Based Intervention for Intimate Partner Violence (IPV) Survivors in Sweden

**DOI:** 10.3390/ijerph23080993

**Published:** 2026-07-29

**Authors:** Cecilia Strand, Freddie Lymeus, Ann-Sofie Bergman

**Affiliations:** 1 Department of Informatics and Media, Uppsala University, 753 12 Uppsala, Sweden; 2 Institute for Housing and Urban Research, Uppsala University, 753 09 Uppsala, Sweden; freddie.lymeus@ibf.uu.se; 3 Department of Psychology, Uppsala University, 751 42 Uppsala, Sweden; 4 Department of Social Work, Stockholm University, 106 91 Stockholm, Sweden; ann-sofie.bergman@socarb.su.se

**Keywords:** intervention development, iterative design, participatory action research, intimate partner violence, group support, women’s shelter

## Abstract

**Highlights:**

**Public health relevance—How does this work relate to a public health issue?**

- With approximately one-third of all women expected to experience physical, psychological, or sexual violence from a current or former intimate partner during their lifetime, IPV constitutes a global public health and human rights challenge.
- IPV is associated with prolonged and complex mental health care needs.

**Public health significance—Why is this work of significance to public health?**

- In many contexts, including Sweden, non-profit women’s shelters constitute a key provider of IPV support, offering a diverse set of services that extend beyond those provided by public care and service providers.
- This project offers practical guidance to actors engaged in intervention development and in expanding non-profit actors’ methodological toolbox and ability to support IPV survivors.

**Public health implications—What are the key implications or messages for practitioners, policy makers, and/or researchers in public health?**

- Participatory action research combined with iterative design approaches offers an agile and transferable framework for intervention development in close collaboration with users and end-beneficiaries.
- Co-creative intervention development, guided by epistemic equality between researchers and community stakeholders within a well-defined value system and collaborative process framework, ensures project integrity as well as the feasibility and contextual relevance of its outputs.

**Abstract:**

There is a lack of step-by-step guides for developing evidence-based interventions through close collaboration between researchers and practitioners that emphasize genuine co-creation. The purpose of this paper is to provide a detailed description of the five-year participatory action research- and iterative-design-inspired development process that resulted in a 12-week group-based intervention to support reorientation and post-traumatic growth among intimate partner violence (IPV) survivors. This paper presents a case study based on an analysis of meeting agendas and notes, observations, successive versions of the intervention, and interviews with staff at partnering women’s shelters and female victim-survivor intervention participants, conducted from 2021 to 2025. This study offers future intervention developers three actionable recommendations: (1) invest in relationship building and establish a collaborative project culture characterized by epistemic equality between academic researchers and practitioners, manifested in non-hierarchical decision-making; (2) create a purpose-built framework for genuine collaboration and co-creation, like the one presented in this project, which combines participatory action research and iterative design approaches; and (3) ensure a shared vision and a formalized agreement regarding future uses and rights connected to the intervention. By offering key transferable recommendations, this paper contributes to teams embarking on intervention development alongside end-beneficiaries and community stakeholders in women’s shelters and beyond.

## 1. Introduction

Intimate partner violence (IPV) is defined as physical, psychological, sexual, economic, or material violence perpetrated by a current or former partner or sexual intimate. Approximately a third of all women globally will be victimized by a current or former male partner during their lifetime, making IPV a serious public health and social policy challenge across several contexts. While men can also be victimized and violence can occur in same-sex relationships [1,2], the high prevalence of male-perpetrated IPV against women has led to its being described as a global pandemic [3]. Furthermore, besides being more prevalent, male-perpetrated IPV is more likely to cause serious injury and to impact the victim’s sense of bodily safety to a higher degree [1,2].

Beyond sheer volume, the complexity of the negative effects on victim-survivors poses a significant challenge for communities and societies around the world. While many governments have expanded services, few, if any, are able to meet the needs, and non-profit women’s shelters remain a crucial provider of support services.

Sweden consistently ranks high in gender equality, yet it still has one of the highest IPV rates among the 28 EU member states [4]. While successive Swedish governments have expanded services to meet the needs of IPV survivors, Swedish non-profit women’s shelters (kvinnojourer), informed by feminist ethics of care, continue to be a key provider of support, particularly psychosocial support aimed at facilitating post-traumatic growth. This article details the development of a new psychosocial method—the group intervention *Strength to Move On* (Kraft att gå vidare)—co-created by a team of researchers and Swedish women’s shelters. Through an analysis of key transferable lessons, the article offers future intervention developers an outline for a collaborative model and workflow.

### 1.1. Background and Context of the Project

Male-perpetrated IPV against women has complex, long-lasting negative effects on victim-survivors and their children. Multiple studies, both international and Swedish, have found that many IPV survivors experience mental health symptoms, including post-traumatic stress disorder (PTSD), anxiety disorders, depression, and suicidal ideation [5,6]. Many also develop somatic symptoms due to trauma [7]. These mental and somatic symptoms and health risks often persist well beyond separation from the perpetrator. Furthermore, post-separation abuse, through continuing threats, harassment, and intimidation, is common and often exacerbates the negative long-term impacts. Post-separation economic and legal abuse often constrains access to the financial resources needed to regain control and rebuild an independent life [8]. In short, past and/or ongoing violence affects the survivor’s ability to function across multiple spheres of life and makes rebuilding a shattered self and life after leaving the perpetrator an arduous and complex task. Providing female victim-survivors with adequate support to rebuild their lives after leaving a violent partner challenges societal support systems across contexts, and Sweden is no exception.

Historically, support for IPV victim-survivors was almost exclusively provided by non-profit women’s shelters that emerged from the women’s movement [9,10]. The Swedish women’s shelter movement emerged through the formation of local women-to-women groups, thus displaying similar patterns observed in the US [11], Canada [12], the UK [13], and beyond [14]. Swedish feminist activists, as in other contexts [15], mobilized to address a lack of safe housing and support for victim-survivors, to promote their analysis of IPV against women as embedded in features of hegemonic patriarchy, and to advocate for policy change and increased resources. Beginning in the 1990s, successive Swedish governments launched various initiatives to combat IPV, including criminal law reforms [16], social policy changes, and updated directives for health care providers, social services, and other public actors [17]. From an international perspective, Sweden has emerged as a policy-forward context regarding IPV [18,19]. Despite an incremental expansion of victim services, public health care and social services are still unable to fully meet either the practical needs or the long-term psychosocial support needs of IPV victim-survivors [20,21,22]. Non-profit women’s shelters continue to be major service providers in Sweden [23,24], and across Europe [25]. Women’s shelters play a pivotal role in providing psychosocial support beyond the acute phases of separation, as they can offer support for years in cases of prolonged post-separation abuse.

There are approximately 200 non-profit women’s shelters in Sweden [25], which typically provide a range of informational and practical services, as well as psychosocial support, primarily through individual counseling conducted in person and/or remotely. In addition, some shelters offer safe emergency housing, legal aid, and social activities. Some women’s shelters also offer support groups, ranging from informal, open groups to closed groups that follow a structured intervention format. Despite the multiple group-based intervention formats in use, these have not been evaluated with sufficient scale or rigor to understand their efficacy, mechanisms of change, or when and to whom they should be offered in the context of separation and recovery. Furthermore, some interventions that were primarily developed for social services or health care may lack feasibility or effectiveness in the unique context of women’s shelters. The lack of evidence-based, holistic interventions that address the complex long-term needs resulting from IPV is not unique to the Swedish case; it has also been noted in other contexts [26].

Against this backdrop of unmet psychosocial needs, particularly in relation to addressing the long-term residual impacts of IPV on victim-survivors and a lack of systematically developed and evaluated methods for supporting victim-survivors’ journey in rebuilding their lives, a small group of scholars with interests in intervention development, participatory approaches, and psychosocial aspects of heath, together with a local women’s shelter (the primary project partner) began to explore opportunities for collaboration. What began as an exploratory conversation was followed by a five-year intervention development process that resulted in a manualized group intervention—*Strength to Move On*—with high acceptability and perceived utility, as well as initial evidence concerning its positive impacts on key psychological health domains [27,28]. The intervention development process also yielded a number of valuable, transferable lessons that could inform future development projects in women’s shelters in other countries or in similar non-profit psychosocial care settings.

While the project is situated in the Swedish context, several of the intervention context’s challenges are observable across Europe [25]. Swedish women’s shelters have historically benefited from the enabling political environment in which gender equality has been a prioritized political project [29]. The last decades have, however, seen a move towards marketization with the introduction of profit-driven private actors in the publicly funded welfare system [24,29] and increased competition in the service field of women’s shelters from both commercial and state-run services [9,30]. State- and municipal-level austerity policies, combined with marketization and gender-neutral policies, have significantly reduced available resources, challenging women’s shelters’ autonomy and their ability to continue providing services. New legislation, introduced in April 2024, regulating the operation of emergency safe housing, combined with a complex and prolonged compliance process, posed a significant challenge for non-profit shelters and led to a 59% reduction in non-profit safe emergency housing between 2023 and 2026 [30]. The Swedish shelter context thus shares many of the same challenges observed in other European countries [25,31].

### 1.2. Purpose of the Present Study

The purpose of this paper is to describe and analyze the five-year participatory action research (PAR) and iterative, design-inspired intervention development process of *Strength to Move On*. Specifically, its aims are twofold. First, this study aims to provide future intervention development teams with a comprehensive description of how the multi-phase, co-creative development process was conducted by exploring the craft of intervention development in multidisciplinary teams, with community stakeholders and the experiences of practitioners and participating victim-survivors at the center. Second, we seek to highlight key transferable lessons learned along the way, with the aim of supporting future teams in optimizing their processes and avoiding unproductive detours and delays.

We recognize that our case study is unique and that other intervention developers will inevitably have different aims and constraints. However, during the course of our project, we found that existing models and examples, drawn mainly from clinical and public health fields, were not directly applicable and were of limited use in the context of women’s shelters. In particular, such models tended to take a much more theory-driven, linear approach with less stakeholder involvement than what would be suitable and effective for developing an intervention from the ground up with actors whose practices are heavily influenced by ideological boundaries and resource constraints. To help bridge this gap while respecting the unique and emergent nature of collaborative development projects across contexts, we sought to describe our specific process, summarize the key lessons learned, and ultimately distill them into transferable lessons that we believe hold general relevance across cases.

The remainder of this article is organized as follows: Section 2 introduces the methods and materials, including an outline of the analytical steps that led to our lessons learned and an introduction to the intervention and the three principles that informed its development: PAR, multidisciplinarity, and an iterative design approach. Section 3 describes the four main phases of intervention development, outlining the key activities and objectives of each phase and the lessons learned. Section 4 synthesizes these lessons into overarching lessons considered to be broadly relevant to future in tervention developers, and Section 5 discusses the potential utility and limitations of the analyses and conclusions.

## 2. Methods and Materials

### 2.1. Methodological Approaches of the Present Case Study

In this study, we employed a descriptive single-case study research design, which is particularly effective for exploring contemporary phenomena in real-world settings [32]. Typically in such settings, the boundaries between a phenomenon and its context are not clearly evident, and understanding of the internal dynamics of the case depends in part on analyses of the contextual conditions. This research design allows for both descriptive and exploratory elements, combined as needed to meet the specific study’s needs. The case under study is the development process of the *Strength to Move On* intervention, from its early ideation phase in late 2020 to its release for independent use in Swedish women’s shelters in the spring of 2026.

**Figure 1:**
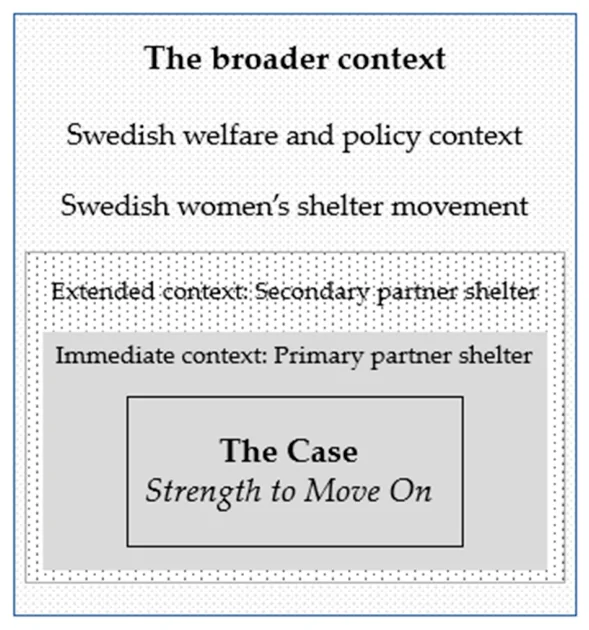
The case embeddedness in multiple contexts.

A descriptive single-case study design allows for a detailed yet holistic exploration of the case—the development of *Strength to Move On*—in its context, which consists of both the primary partner shelter (Uppsala kvinnojour), where the intervention was first developed and pilot-tested, six secondary partner shelters (Kvinnojouren Annfrid, Södertälje; Kvinnojouren Huddinge; Kvinnohuset Västerås; Kvinnojouren Linnéan, Lidköping; Kvinnojouren Ellinor, Linköping; and Kvinnojouren Mira, Nyköping) that implemented the intervention in a more developed form, and the broader field of Swedish women’s shelters that connects this work to the particular history and ideology, tradition, and framework of surrounding actors and policies. That is, we considered the case context to be multi-layered (Figure 1).

The motivation for this study is to explore the intervention development process and ultimately identify and share the factors and processes that contributed to the output. Thus, this study broadly aligns with Yin’s [32] proposed rationales for descriptive case studies, which explore “how” and “why” questions about a particular social phenomenon. In particular, the study sought to highlight how the intervention was developed, how different stakeholders were engaged in the process, and how participatory approaches informed its development. Furthermore, this study sought to understand the causal links between decisions made throughout the intervention development and the end product—*Strength to Move On*.

The original team of six researchers had an a priori interest in community-based intervention development and was guided by the theoretical proposition that intervention development can benefit from participatory approaches, as involving key stakeholders is likely to promote the intervention’s feasibility, acceptability, and relevance to the target group [33]. Furthermore, we assumed that participatory development could foster a sense of ownership among stakeholders, empowering them to sustain and institutionalize the intervention after the development process.

**Figure 2:**
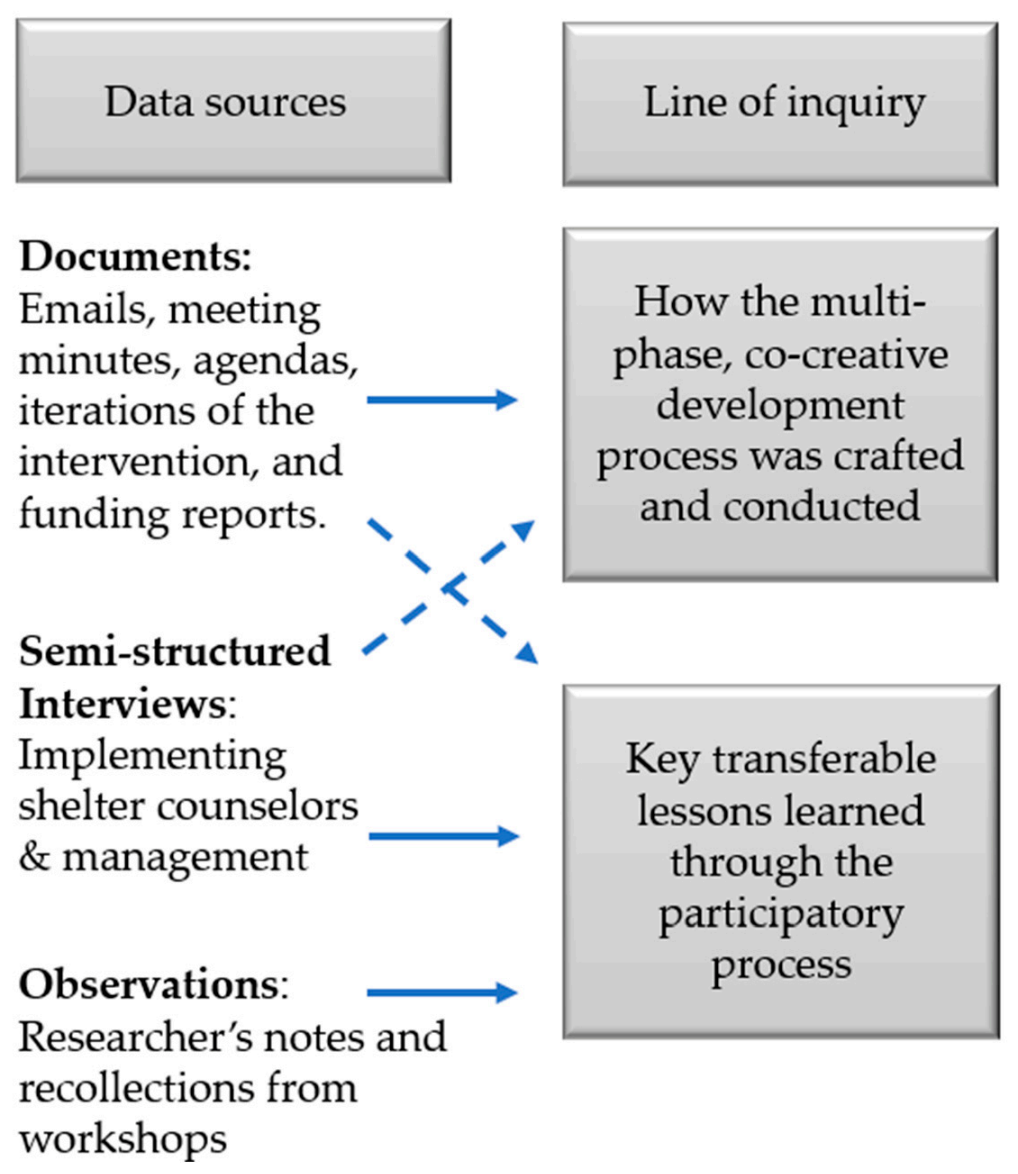
The case study’s data triangulation approach, with solid lines indicating primary data sources, and dashed lines representing secondary data sources.

Aligned with case study methodology, we drew on multiple data sources to conduct the analysis through data triangulation (Figure 2).

We drew on three types of data sources: documents, semi-structured interviews with shelter staff and management, and observations made during intervention development workshops in project phase 2. Figure 2 visualizes the use of the different data sources and their relation to the line of inquiry. Together, the data sources captured the multi-phased intervention development timeline and the complex development process from multiple perspectives, including the multiple rounds of brainstorming and design sessions, testing in shelter environments, discussion, critique, and redesigns of different parts of the intervention over the five years.

Document analysis was primarily used to clarify the timeline of various activities and to reconstruct the four phases of intervention development through analyses of meeting agendas and minutes from research group meetings and meetings with shelter partners, including emails and records of various iterations of the intervention. Documents directly related to the deliberation and co-creation processes were also used, along with semi-structured interviews and observation data, to triangulate key lessons learned from the participatory practices. The observations consisted of recollections, notes, and materials from the workshops where various intervention components were introduced, tested, and discussed. Although all three data sources were used to identify and formulate key transferable lessons learned, 13 semi-structured interviews with counselors and shelter management at seven shelters implementing the intervention formed the core of the data set for this part of the analysis.

The interviews with intervention implementers focused on exploring their experiences of working with the intervention, including participant recruitment, the shelters’ organizational preparations, the group leaders’ individual preparations before and during the intervention period, introducing the intervention to participants, leading a group, observations regarding the participant’s engagement with the intervention’s activities, the use and perceived usefulness of the manual and instructions, and the intervention’s compatibility with the shelter’s existing workflows, value systems, and resource constraints. In addition, the interviews actively solicited constructive feedback on the intervention’s components and overarching structure. The counselor interviews lasted between 50 and 75 min and were conducted one to three weeks after they completed a *Strength to Move On* group. The interviews were analyzed using a directed approach [34]; that is, the analyses were pre-focused on counselors’ explicit statements as they related to the intervention’s (a) feasibility, (b) acceptability, (c) perceived helpfulness, (d) problems identified and solutions proposed with the overarching design and individual exercises, and (e) reflections about the development process. Throughout intervention development, counselors’ interviews were formative, functioning as source material guiding continuous development and adjustment. In this case study, interviews were also treated as arenas for tracing co-creation; thus, the analysis focused on identifying and tracing the counselors’ suggestive statements and their subsequent impact and integration into the intervention.

In addition to the counselor and manager interviews described above, the intervention development process also involved semi-structured interviews and surveys conducted directly with the end-beneficiaries, that is, IPV victim-survivors participating in a *Strength to Move On* group. These sources were used to understand and evaluate different iterations of the intervention’s impact, generating results reported elsewhere; however, they were not used as primary data in this case study, which focuses on the development process rather than on the conceptualization and evaluation of the intervention itself.

### 2.2. Case Study, Part I: Introduction to the Intervention Strength to Move On

*Strength to Move On* is a manualized group intervention for women IPV victim-survivors who struggle to rebuild their lives after separation. A complete account of *Strength to Move On*, including its eclectic health-promoting approaches and related conceptual underpinnings, is presented elsewhere [27,28]. Here, we focus on the intervention’s guiding principles and formal structure as background to the main parts of the paper, which describe and analyze the development process.

*Strength to Move On* was developed to align with the ideological, organizational, and staffing conditions of Swedish women’s shelters and to be implementable without investments in specialized training or materials. The intervention consists of practical exercises inspired by therapeutic and health-promoting traditions adapted to the shelter context and users (shelter staff and IPV victim-survivors). Intentionally, none of the exercises are psychoeducational, directly address processing past experiences of violence, or provide explicitly IPV-related coping strategies—such themes and practices were already relatively well represented in shelters’ individual counseling and psychosocial services. Instead, *Strength to Move On* was purposefully built to be forward-looking with the aim of supporting reorientation, strengthening victim-survivors’ personal resilience strategies, and facilitating post-traumatic growth by promoting greater connection with personal values, including valued social connections, places, and activities; strengthening stress management and self-care habits; and cultivating feelings of mental and embodied autonomy, agency, and strength. The intervention is structured as a psychosocial support program that supplements, rather than replaces, any medical or psychological treatments that participants may need.

The intervention uses a closed-group format, typically consisting of 5–8 participants, and is led by two shelter counselors. The 12-week intervention comprises six modules, each with a 2.5 h group meeting at the shelter followed by a two-week period of individual integration and “homework” that aims to extend engagement with the group meeting’s themes and promote the adoption of relevant daily-life habits. The rationale behind a bi-weekly structure is twofold. First, many IPV victim-survivors share child custody with the perpetrator and would have difficulty attending group meetings every week. Second, it gives participants more time to process and engage with the group meeting’s material before additional content is introduced.

### 2.3. Case Study, Part II: Guiding Principles for the Intervention Development Process

The intervention development process drew initial inspiration from PAR and multidisciplinary collaboration. Iterative design principles emerged in response to evolving project needs. These three foundational principles, combined, form a comprehensive framework for intervention development in close collaboration with users and end-beneficiaries.

#### 2.3.1. Participatory Action Research

Several studies offer step-by-step guidance on intervention development in the clinical [35,36,37,38] and public health fields [39,40], but we considered them of limited use for crafting a genuine co-creative development process in shelter settings with IPV counselors and victim-survivors (also see [41,42,43]). Given the project’s vision of co-creation in close collaboration with community stakeholders, PAR provided useful guiding principles.

PAR has historical roots in development studies [44]. It is not a set of specific procedures to follow, but rather a philosophy and approach for how to conduct projects, including research [45]. It distinguishes itself from more traditional research methodologies through its foundational emphasis on engaging in actions aimed at improving the world, guided by continuous reflection in close collaboration with stakeholders [46]. PAR thus centers emancipatory collective reflective inquiry, which researchers and other project participants undertake together to understand and improve practices [45,46]. PAR is characterized by (1) its aim to enable action through a repeating reflective “corkscrew” of data collection, reflection, and action, (2) its ambition to deliberately share power between the researcher and the researched; and (3) its resistance to extractive practices whereby information is removed from its contexts, as well as in the active involvement of community stakeholders in the research process and outcome (2006, p. 854) [46]. Ultimately, PAR is focused on epistemic equality and the co-creation of knowledge and new solutions that the community identifies as important and useful.

#### 2.3.2. Iterative Design

Iterative design approaches stem from the fields of human–computer interaction and informatics. An iterative design process proceeds through repeating cycles of testing, review, and refinement [47,48]—a workflow that fits well with the PAR approach described above. Iterative design processes have traditionally tended to be designer-centered, where designers generate concepts and design features based on research and identified needs, followed by user testing and necessary adjustments. By merging iterative design approaches with PAR, the project adopted a participatory iterative design [49], whereby the design process is reimagined as a team effort that equally involves community stakeholders (i.e., women’s shelter staff, IPV victim-survivors) and “designers” (i.e., researchers), in addition to serving as a tool for continuous learning and evaluation [50].

An iterative design approach embraces multiple cycles of testing and refinement, continuing until the design meets the user’s needs and expectations, thereby requiring a high tolerance for repetition and defying fixed timelines for reaching expected targets and results. It is a highly flexible, adaptive process that enables developers to pivot and make informed decisions based on empirical evidence generated through multiple rounds of testing, rather than on assumptions [51]. Clinical intervention development models sometimes refer to iterative design principles but typically take a more linear approach, with development theory-driven, leading to a pilot test followed by one round of revisions [35,36,39]. Iterative design approaches have been observed in a number of public health studies [37,52,53,54,55].

This project involved mutual learning with the intended users, that is, the Swedish women’s shelters and IPV victim-survivors, to achieve high intervention acceptability and feasibility. We drew on the tools available through iterative design to capture user experiences, such as the think-aloud method, which captures cognitive processes and experiences as users engage with a prototype [56], and post-task interviewing [57]. Iterative design methods were combined with focus group interviews with shelter staff [58,59], conducted during the initial intervention development phase, to collect and systematize the practitioners’ insights.

#### 2.3.3. Multi-Disciplinarity and Researcher–Practitioner Collaboration

Several authors have argued that interventions are best developed in multidisciplinary teams and in close collaboration with practitioners and the intended end-beneficiaries [35,40,60,61]. Wight et al. argue that “such coproduction maximizes the likelihood of intervention effectiveness by improving: the fit with the target group’s perceived needs and thus acceptability; practicality; evaluability, including the theorising of causal pathways; and uptake by practitioners and policymakers” all of which were key aims of our intervention development process (p. 520, [43]). With participating researchers spanning multiple academic disciplines and partnering shelter staff representing a heterogeneous mix of educational backgrounds and specialized expertise, as well as a deep experiential understanding of IPV victim-survivors’ needs, the intervention development team embodied the concept of multidisciplinarity. The intervention development process repeatedly benefited from the multitude of perspectives that emerged in this collaborative, creative, and critical environment.

## 3. Co-Creation in Close Collaboration with Community Stakeholders

With the aforementioned inspirations and approaches in mind, the following sections cover the five-year intervention development process, paying special attention to what we believe are key, transferable lessons.

### 3.1. Phase I—Relationship Building and Need Exploration

The Swedish shelter system has a strong tradition of providing flexible, holistic support to IPV victim-survivors across all phases of coping, separation, and reorientation, as well as of working with empowerment within a feminist ethics-of-care framework. Furthermore, non-profit women’s shelters remain relatively free from the constraints of linear service production and bureaucracy that mark surrounding public actors [62]. This relative freedom allows shelters to pursue new collaborations and projects independently. Following an internal preparatory process, the researchers contacted the primary partner shelter to discuss a joint project to strengthen and promote health among IPV victim-survivors. Inspired by PAR, the first phase involved relationship building and the exploration of mutual interests, needs, and constraints. Through several open-ended exploratory discussions between the research group and primary partner, documented in meeting minutes and emails, involving not only counselors and the shelter’s managing director but also its executive board, a team emerged, driven by a desire to address the lack of evidence-based interventions specifically targeting the long-term impacts of IPV that can persist well beyond separation from the perpetrator. The researchers applied for seed funding from the university to develop a research project. The application documents described a vision for an intervention that would promote victim-survivors’ strengths and support positive growth through a series of practical exercises, drawing on established therapeutic and health-promoting traditions. The partnership between the researchers and the primary shelter was formalized in a written agreement that specified the shared plans and the respective roles and contributions of both parties for the one-year funding granted at this stage.

#### Key Lessons in Relation to Relationship Building

In hindsight, it cannot be overstated how important it is to establish a solid understanding of each other’s priorities, needs, non-negotiables, and resources and to formulate a shared vision when working in a multidisciplinary team comprising both academics and practitioners. In this case, the shared vision was an intervention that aligns with the key values and constraints of the shelters, supplements rather than replaces their existing support services, and expands their ability to support IPV victim-survivors’ long-term reorientation and growth, backed by rigorous efficacy, feasibility, and effectiveness testing. Grounded in PAR principles, the relationship-building process was primarily oriented toward understanding the shelter’s experiences and needs and developing a shared view of how research processes could be integrated into the shelter’s normal operations and priorities, ultimately benefiting the project partners and the target group. This foundational alignment, in terms of whose priorities guide the development of interventions, served the project well. However, it later became evident that the shared vision should have encompassed eventual use, future fair rights, access to the intervention, and a formal agreement regarding the right of use.

### 3.2. Phase II—Collaborative Intervention Design and Small-Scale Piloting

With seed funding secured, the process of jointly crafting an intervention could begin. The goals for the second phase were threefold: firstly, to co-create, and test potential intervention components as well as create an intervention structure reflective of the needs of both the shelter and end-beneficiaries, that is, IPV victim-survivors; secondly, to complete a pilot study of the acceptability and feasibility of the intervention’s concept, components, and structure; and thirdly, to secure long-term funding for continued intervention development and larger-scale evaluation.

The initial intervention development process was organized as a series of seven workshops, attended by the research team and representatives from the primary partner shelter, including counselors, a communications officer, and the managing director. The workshops were informed by PAR and an iterative design approach, which provided a clear structure for partners to collaborate on the intervention design [49]. The process was organized as follows:

Workshop 1: This workshop examined various exercise types designed to address the needs of IPV victim-survivors. Exercise types broadly fell into four main categories: (a) bibliotherapeutic practices and expressive writing, (b) mentalization, (c) bodily activation, and (d) meditation and mindfulness. Additionally, the workshop participants specified a one-year work plan that aligned with the research project’s funding timeline.

Workshops 2–3: These two PAR-inspired two-day workshops consisted of presentations and deeper analysis of some of the main types of IPV and their impacts and impact mechanisms, contributing to the attainment of a shared understanding of the complex and heterogenous needs of victim-survivors, followed by the presentation of potential intervention components, that is, specific exercises that could address one or more of the identified needs. Contending exercises had been identified through the researchers’ review of existing therapeutic and health-promoting interventions targeting related needs, some of which had previously been applied with IPV victim-survivors and other relevant vulnerable populations. At the workshops, all exercises were tested together by the entire project team, that is, both researchers and primary partner staff. The workshops can best be described as small exercise laboratories, where the think-aloud and post-task interview methods inspired the approach for capturing immediate feedback from the multidisciplinary researcher–practitioner team.

Workshop 4: Building on insights from the previous workshops, this session focused on establishing a preliminary sequence of exercises considered for inclusion in the intervention to date and on developing an overarching intervention framework by discussing the conceptual relationship between exercises. This workshop also explored suitable modalities for evaluating this first version of the intervention.

Workshop 5–7: Over three full-day workshops spaced one month apart, additional exercises were introduced for consideration, further diversifying and expanding the pool of candidate exercises. The workshops were characterized by genuine co-creation, seeking to merge researchers’ analyses of existing intervention theory and evidence-based practices with the shelter staff’s in-depth knowledge of the target group and of what would be feasible from a shelter’s perspective. Each dry run of a new potential exercise idea was followed by a multidisciplinary review of the exercise’s merits, drawbacks, and requirements for further adaptation to fit the target group’s needs, the shelter context, and resource limitations. The shared experience of discussing and testing the exercises and gaining a tangible, personal sense of their potential execution and impact contributed greatly to a common understanding of the intervention and allowed the counselors to deepen their understanding of the theoretical background and rationale for the exercises under consideration.

In parallel with developing the initial draft intervention’s general structure and individual components, the researchers secured the ethical approval needed to pilot the intervention with IPV victim-survivors (The Swedish Ethical Review Authority, 2022-02099-01, later supplemented by several approved amendments) and submitted several research project applications to fund a larger-scale evaluation beyond the pilot. Although two of the six researchers left the project during phase 2, the pilot began as planned. As the researchers left, we replaced the exercises they had introduced into the intervention.

#### 3.2.1. Piloting the First Version of the Intervention

With the ethics approval in place, the project team proceeded to pilot the first version of the intervention. Eight adult women were recruited from the primary partner shelter’s existing clientele to participate in *Strength to Move On.* These women were all at least 12 months post-separation and had either previously received individual counseling or were currently receiving it. However, they all still struggled in different ways with the negative long-term impacts of past victimization and were motivated to participate in a group-based intervention for IPV victim-survivors. The pilot group was led by two counselors who had been involved in developing the intervention and had prior experience facilitating IPV support groups. The counselors also served as intermediaries in the research by providing participants with study information, answering questions (when needed after communicating with the researchers), collecting consent forms, and scheduling participants for interviews with the researchers. In addition to the shelter staff’s selection process, which determined who was approached to join the pilot group, all interested women completed a survey-based screening that was managed and evaluated by the researchers. This survey used established psychometric screening instruments to ensure the possible participants had no severe current mental health symptoms and no indications of risk for serious mental illness at the beginning of the pilot. Participants signed informed consent forms and agreed to be interviewed as part of the evaluation of the intervention. The group was heterogeneous in terms of age, socioeconomic and educational backgrounds, as well as protective status and experiences of violence, which allowed the diversity of perspectives on the intervention to be maximized.

Participants’ experiences with the intervention were documented through a series of semi-structured interviews conducted using a digital meeting application in the days following each of the six group meetings and one month after the final group meeting. The study protocol also included a one-year post-intervention follow-up of the participants. The interviews explored participants’ experiences with each specific exercise and, more broadly, with participating in the group, as well as any changes in psychological well-being they attributed to the intervention. The pilot study thus used a research design that not only allowed the overall experience of participating in the intervention to be evaluated but also assessed how each exercise was received by the users. All interviews were transcribed and analyzed using a directed approach [34]; that is, the analysis focused on participants’ explicit feedback on the exercises’ acceptability, relevance, impact, perceived helpfulness, and clarity of instructions. Note that we mention these participant interviews here as part of our description of the development process, not as a method or data source in the case study of the development process of the intervention.

As the primary partner’s staff were involved in the entire intervention development process—including the conceptualization, construction, and internal dry-runs of exercises—the pilot was not preceded by any additional training for implementers. Implementation was, nevertheless, guided by regular meetings between the researchers and implementing staff, during which implementers’ observations were collected, emerging issues were discussed, and agile problem-solving was undertaken. This led to several minor changes being made to the intervention’s exercises and structure, requested by the implementers throughout the pilot. In line with previous intervention research [35,36], which highlights the importance of capturing and examining implementers’ experiences during pilot studies, the pilot study included semi-structured interviews with the counselors who led the group. Interviews focused on their experiences working with the materials, introducing and explaining exercises, guiding sharing and reflections triggered by the exercises, their observations of participants’ engagement with the exercises and the group, their perceptions of the intervention’s relevance to meeting participants’ needs, as well as its positive and negative impacts on participants. The interviews also explored their perceptions of feasibility in relation to the shelter’s overall operations and resources. The PAR-inspired agile and continuous adaptation throughout implementation was valued by the implementers.

“*We reviewed it (referring to an exercise) and talked about “what do we think about this,” and then we tested it, because, well, the physical exercises need to be part of the muscle memory, and such. Then we reasoned, “Well, what do we think, maybe it is better this way instead, and it might be good to switch it up like this?” Afterward, we sent written feedback on it, and there has been an incredible responsiveness from your side, where our suggestions were taken seriously and later implemented*”

(Counselor, primary partner, pilot)

Furthermore, in an interview with the implementing counselor and management at the primary shelter, it became clear that the intervention development process and its implementation both challenged staff and management and created valuable new learning opportunities for the organization and its staff.

“*So I think we have kind of grown into this together. On one hand, we (referring to the shelter staff) have trained at the shelter together before, and then when you do it together in the group… and I have only positive experiences of this because I think everything has been so much more.… It has exceeded all expectations, this entire project. What I also find interesting is to see how differently the intervention has resonated with different women. So I have nothing but positive things to say, from a personal perspective, from a professional perspective, and also for the women we meet, and for us as a team, I would like to add. It has been an experience of growth!*”

(Shelter manager, primary shelter, pilot)

Before the pilot study was completed, the research team was awarded a three-year research grant to continue developing and evaluating the intervention. The grant enabled both scholars and primary partners to commit to an extended timeline and expanded scope.

#### 3.2.2. Key Lessons for Collaborative Intervention Development

A number of lessons can be drawn from the second phase of the project. First, guided by PAR, practitioners’ direct involvement in the early stages of conceptualization and development fostered mutual respect and appreciation for the team’s diverse yet complementary expertise. By recognizing practitioners as key knowledge-bearers and experts, the development process was, by default, centered on the shelter staff’s intimate knowledge of IPV victim-survivors’ needs and typical challenges. Second, by engaging both shelter counselors and management, the nascent intervention was designed with feasibility in mind. That is, the intervention’s features were designed from the outset to fit the specific ideological, organizational, physical, and economic context of a women’s shelter. Third, through the PAR-inspired development process, the primary partner shelter co-owned the process and had a real stake in it and its outcome.

### 3.3. Phase III—Extensive Revision Work, Agile Evaluation Protocol, and Manual Development

With basic feasibility established through the pilot and longer-term funding in place, the development process was scaled up and entered a new third phase under the research project titled *Strength to Move On—an intervention for strengthening IPV survivors*. The overarching aims of the research project were to further develop the intervention while implementing and evaluating its effects on IPV victim-survivors; to explore its complementarity with, and contribution to, existing psychosocial practices in Swedish women’s shelters; and to establish conditions for the continued dissemination and use of the intervention after the project’s completion.

There must be sufficient evidence of acceptability and potential benefits to justify investing more time and resources and to expose a larger number of users to an intervention [43,63]. The process typically involves smaller-scale data collection to demonstrate that the intervention is needed, practical, beneficial, and working as intended and that it poses no serious risks. The supervised implementation protocol during the pilot study—consisting of regular meetings with the shelter staff involved and interviews with IPV victim-survivors—had already established that the intervention was broadly acceptable: the exercises generally made sense to the leaders and participants and worked as intended. Ultimately, all pilot group participants completed the 12 weeks. They reported overwhelmingly positive experiences and no concerning negative experiences. Note, however, that participant interviews and formal analyses of intervention processes and outcomes fall outside the scope of the current case study.

The third phase commenced by engaging with the pilot study’s rich interview data. Because interviews were conducted after each of the six group meetings, the pilot study generated rich descriptions of engagement with the exercises and group processes. A systematic analysis of the interview material identified intervention components, such as exercises, instructions, and procedures, that required additional attention and redesign. The primary critical feedback was insufficient time to complete the exercises, resulting in rushed or skipped exercises and merging the much-needed and cherished coffee break with intervention work. The feedback also called for simplifying and shortening the instructions for some exercises, and it became clear that the intervention was perceived as lacking clear internal logic and an easily discernible structure or progression between the modules. Most participants also requested more time to socialize and opportunities to get to know the other participants better. The analysis and redesign process could also draw directly on shelter counselors’ privileged position as observers of participants’ engagement with the different exercises and the evolving group dynamics and processes. Counselors were thus able to provide an additional layer of understanding of how certain intervention components needed to be redesigned.

The combined findings were addressed in several design meetings, during which exercise instructions were simplified, some exercises were moved between modules to improve internal coherence, and the progression logic was clarified. Aligned with PAR principles, we prioritized feedback from counselors and IPV victim-survivors over any theoretical assumptions or therapeutic conventions.

#### 3.3.1. *Strength to Move On*, Version 2.0, and the Introduction of a Mixed-Method Evaluation Protocol

Funding for continued intervention development and evaluation enabled the introduction of a more comprehensive research design and evaluation protocol. The research design involved collecting quantitative data through surveys before, during, and after the intervention period. These surveys targeted common mental health symptoms (the primary outcome), general psychological health, and presumed mediators, including self-compassion, engaged living, and group cohesion. The study design also included semi-structured qualitative interviews with group participants at the beginning, middle, and end of the 12-week intervention period. However, the results of the analysis of these data are presented separately [26,27] as they fall outside the scope of this article.

Similarly to before, we interviewed the shelter counselors after they had completed the 12-week intervention. When exploring implementers’ experiences, interviews covered the same topics as in the pilot study, as well as questions concerning participant recruitment and organizational preparations. The interviews also explored implementers’ direct observations of how participants engaged with the intervention content and with one another, as well as their perceptions of the intervention’s potential impacts on participants.

#### 3.3.2. A New Round of Small-Scale Testing and Exploration of the Role of the Group

Given the intervention’s significant changes based on pilot study findings, we decided to conduct another round of small-scale testing at the primary partner shelter before scaling up the evaluation and inviting additional shelter partners. This additional small-scale testing was further motivated by an unexpected secondary finding uncovered in the pilot study’s interview material. The interview transcripts were filled with unprompted references to the group’s role as a key contributor to experienced benefits, overall satisfaction, and the perceived helpfulness of the intervention experience. The participating IPV victim-survivors highlighted the group’s importance as an enabling environment for engaging with the exercises, for making sense of survivorship experiences collectively, for exploring paths to both collective and individual agency and autonomy, and for alleviating loneliness and isolation. The group was repeatedly referred to as a safe space and, for many participants, a key enabler of engagement with the challenging material.

With group dynamics identified as a key enabler, *Strength to Move On*, version 2.0, was tested in two groups that differed in size (*n* = 5 vs. *n* = 11) and were led by different pairs of counselors. Analyses of variation in group size indicated that the larger group experienced higher attrition rates and that larger group size was associated with lower intervention satisfaction. Some participants reported feeling overwhelmed and reluctant to contribute to the reflection rounds. Moreover, interviews with participants affirmed the pilot study finding that the perceived benefits of the intervention were deeply intertwined with the experience of meeting other victim-survivors in the group. In particular, many participants expressed appreciation for the intervention’s clear forward-looking orientation, that is, being among fellow victim-survivors who shared similar challenges without focusing on past experiences of violence, on the perpetrators, or even on ongoing post-separation abuse. Instead, the group was described as a safe and uniquely supportive environment, from which participants would engage in the intense self-reflection and reorientation processes built into the intervention.

However, although the intervention had been revised immediately before this study phase, it had to be extensively redesigned again when a research group member unexpectedly left the project. To avoid problems related to authorship, copyright, and ultimately the long-term future of the intervention, 11 exercises that could be tied to the departing researcher were replaced. This was disruptive, as some exercises had to be replaced midway through implementation. The disruption, however, allowed for a candid and unrestrained overhaul of the intervention. The redesign therefore involved not only several new exercises but, most prominently, a more coherent structure that sought to enhance the therapeutic progression and to further strengthen beneficial group processes across both exercises and modules. Following these changes, the redesigned modules 1–3 mainly emphasized self-exploration, in which participants mapped their existing resources and mental and bodily autonomy, whereas modules 4–6 placed greater emphasis on strengthening self-care habits, agency, and value orientation. Different stress- and anxiety-reducing techniques were introduced throughout the intervention, with gradually increasing complexity and challenge.

*Strength to Move On, version 3.0,* embodied rationales similar to those of prior versions, albeit with many new exercises and a more developed internal logic and progression. However, the unexpected disruption midway through the implementation round also disrupted the project’s PAR-inspired work processes. Because material had to be replaced immediately, a more streamlined development process was necessary, in which replacements were conceived and introduced into the intervention by the research team, rather than emerging from a co-creation process; that is, the project team had to accept a pragmatic deviation from the PAR principles. This disruption of the collaborative process, implementation, and evaluation posed a challenge to the shelter counselors, who had to adapt to partly unfamiliar and untested material on very short notice. Overall, the experience highlighted the value of the project’s PAR-inspired collaboration and iterative design approach, where the workflow naturally provided both researchers and shelter staff with opportunities to shape each exercise, develop a sense of how it would be executed and its potential impacts on participants, formulate instructions that fit the situation and target group, and determine how much time would be needed to complete the exercise without stress. The unplanned and fast-tracked changes created a profound awareness of the many benefits, and, when reflecting on this experience, the shelter counselors expressly stated that it helped them recognize the value of being an integral part of the development processes.

#### 3.3.3. *Strength to Move On*, Version 3.0, and New Partners

The project plan entailed scaling up and out by introducing the intervention to several partnering shelters. Grounded in the iterative design approach and with the intervention significantly revised during and after the last round, the primary partner conducted a new round of *Strength to Move On* in order to carry out a complete and rigorous evaluation of the revisions before introducing it to new partnering shelters.

The results from this round indicated that *Strength to Move On* 3.0 remained highly acceptable and subjectively beneficial. Furthermore, the newly introduced exercises and structure were well received and required only minimal adjustments. Semi-structured interviews with the shelter counselors welcomed the explicit logic and clearer internal progression across the exercises and modules. The counselors reported that the intervention’s gradually increasing level of difficulty and complexity, introduced to participants over the 12 weeks, facilitated individual and collective growth. Progression across the 12 weeks had emerged as an unmistakable feature.

This round of implementation involved new counselors who had not been part of the intervention’s development or earlier rounds at the primary shelter. Interviews with counselors new to the project revealed a blind spot—a lack of clear instructions on organizational and counselor preparations before group meetings, as well as guidance on leading specific exercises and supporting key group processes such as trust and intra-group communication. The introduction of novice implementers highlighted that the primary partner counselors’ accumulated tacit knowledge—for instance, how to prepare for group meetings, divide tasks between the two leaders, moderate group discussions, and create supportive social and physical space for self-exploration. With additional new shelters scheduled to test the intervention, it became apparent that a comprehensive implementer’s manual was needed, in addition to the printed participants’ workbooks and planned regular digital meetings with the researchers. With the realization that future shelters would have few opportunities to receive personalized guidance on- demand, creating a manual became a priority.

The manual drew heavily on semi-structured interviews conducted with primary partner counselors after each previous intervention round and notes from check-in meetings. The manual included sections on (a) recruitment considerations for creating an enabling group environment and pre-implementation preparations, (b) an introduction to the intervention structure and modules, as well as the research and logic underpinning each exercise, (c) practical advice for executing the exercises, and (d) techniques for supporting the emergence of conducive group dynamics. The primary shelter was also asked to read and comment on the draft manual text before it was introduced to new partner shelters.

#### 3.3.4. *Strength to Move On*, Version 3.0, and Naturalistic Testing

With version 3.0, the intervention had reached sufficient stability in its overall structure and individual components, and it was supported by sufficient implementation guidance in the form of a manual, enabling its introduction and evaluation in previously unaffiliated shelters. While development processes continued, the project’s emphasis shifted from a primary focus on intervention development to a stronger focus on broader implementation and evaluation. Introducing the intervention to new partner shelters provided an opportunity to test it under naturalistic conditions, that is, in real-world settings with minimal interference by the researchers. The new implementers, who had no prior training or experience with the intervention, relied heavily on the newly developed manual, testing it and thereby generating input into its improvement. We once again started small. In addition to testing the manual’s ability to provide adequate guidance to implementing counselors, this first round of testing in secondary partner shelters also allowed for more evidence on implementers’ and participants’ experiences and perceived benefits, as well as any unforeseen challenges and risks in shelters with no prior stake or bias related to *Strength to Move On*, to be captured.

The recruitment of new partners coincided with the introduction of the previously mentioned legislation, which mandated that Swedish shelters apply for accreditation and complete extensive compliance work to keep their emergency safe housing open [64]. Although many shelters signaled interest, reported having organized support groups in the past, and were convinced of the benefits of working with groups, they lacked sufficient staff resources to participate in the project while completing compliance work. The intervention, version 3.0, was, however, introduced to a handful of interested shelters during a full-day meeting that covered the project’s background, an introduction to the ongoing research, preliminary findings from the preceding rounds of implementation, and a selection of illustrative exercises.

Two shelters had sufficient staff capacity and became the first secondary partners to join the project. Their implementation served as a test bed for the intervention under naturalistic conditions. The implementing counselors had access to support from the researchers via email and online meetings as needed, but were instructed to rely on the manual as their primary source of guidance. Counselors were also asked to document any unclear instructions throughout the intervention’s implementation and to share suggestions for improvements to the materials and manual. The evaluation protocol of post-intervention interviews with counselors and interviews and surveys with participants remained the same. The results from the secondary partner shelters’ implementation of *Strength to Move On*, version 3.0, broadly confirmed previous conclusions regarding feasibility, acceptability, perceived benefits, and satisfaction. One shelter had previously offered a shorter group intervention and had developed clear internal guidelines for recruitment. The shelter generously shared its key insights from past recruitment, recruitment interview guide, and assessment criteria for determining whether a victim-survivor was ready for group work, thereby greatly contributing to the recruitment section of the manual.

*Spontaneously, I think the most important thing I need to find out during the interview (referring to the shelter’s interview of potential participants) is to find out where she is in terms of ambivalence in relation to the perpetrators. Because if she is even slightly ambivalent, it will not work.*

(Counselor, secondary shelter partner, version 3)

Of note, the interviews with participants and shelter counselors revealed that some exercises requiring participants to explore aspects of connectedness, agency, and personal boundaries through physical movement needed more space than some shelters had. Consequently, optional variations of these exercises were developed and introduced into the manual to provide alternatives for shelters with limited office space. The interview data prompted only minor revisions to the manual and some exercises.

#### 3.3.5. Key Lessons in Relation to Extensive and Repeated Revision Work and Testing

Several key lessons can be drawn from the third phase of the intervention development process. The first lesson is to truly embrace the iterative design approach’s core of repeated design, trial, analysis, and redesign, which entails understanding the value of repeated trials and repetition, even with small variations at times. The intervention evolved with each completed cycle, and each cycle’s emerging findings informed the next. Moreover, this project phase showed the necessity of allowing ample time for multiple rounds of not only initial small-scale testing in the primary developing site but also as part of the process of scaling up and out to implementation in different, increasingly naturalistic contexts. The project team gained valuable insights into the variety of shelter environments, each with its own unique challenges, which enabled further development of the manual to support implementation across diverse settings. The second key lesson is to remain exploratory, agile, and open to re-conceptualizing the structure and content as needed, even in later project phases. Agility was particularly tested during the disruption that followed a researcher’s departure from the project; nevertheless, agility enabled the remaining team to approach the situation as an opportunity to revisit and rethink previous decisions. It should, however, be noted that the disruption could have been avoided if the relationship-building phase had included a discussion of a shared vision of rights of use and a formal agreement, outlining how rights to the intervention should be handled.

Finally, an overarching lesson learned is to continuously monitor the policy field for potential policy changes and assess their implications for the project. The compliance requirements for operating emergency safe housing placed a heavy administrative burden on many shelters, forcing them to prioritize compliance. Although the new legislation certainly affected the primary shelter, it had sufficient resources to remain fully engaged in the project while simultaneously securing its accreditation. As several of the approached shelters reluctantly declined to participate in the project to prioritize compliance work, recruiting new partners proved far more time-consuming than initially anticipated. By continuously monitoring sector-specific policy changes, project teams may pre-empt potential delays by adjusting the timing of some project activities.

### 3.4. Phase IV—Larger-Scale Evaluation of Strength to Move On

The final stage of intervention development should entail collecting “sufficient evidence of effectiveness to proceed to a rigorous evaluation” (p. 520, [43]). Phase IV, the last stage of the development process, therefore consisted of collecting evidence on feasibility and potential benefits across a wider set of partnering shelters. As the intervention had reached sufficient stability in version 3, this stage focused on ensuring that the intervention worked as intended across a wider set of shelters and the IPV victim-survivors they support, and that it was not associated with any unforeseen risks. This phase involved the primary partner, the two secondary partners from phase III, and four new shelters running *Strength to Move On* groups. The new secondary partner shelters introduced additional variability across several dimensions, including economic circumstances, the numbers and stability of their employed and volunteer staff, staff backgrounds and experience levels, the sizes and characteristics of their surrounding cities and uptake areas, and the demographic and socioeconomic compositions of the participating victim-survivors. Some of the new partners were also still engaged in compliance work to keep their emergency safe housing open.

Informed by PAR, the timing of implementation was aligned with the shelters’ preferences and constraints, resulting in five shelters starting at different times and one shelter choosing to run intervention group meetings weekly, rather than every other week, and combine it with free child care for those who needed it. Multiple sites, with varying levels of resources available and varying levels of understanding of the research protocol needed to evaluate the intervention, as well as differences in the timing of the intervention start, posed a significant logistical challenge for the project team. The data collection followed the same protocol as in the preceding rounds. Feedback from implementing counselors was consistently favorable, indicating that the intervention had reached a level of maturity.

“*We have long been convinced of the importance of groups and have wanted to have groups to a greater extent than we have been able to in the past. So this initiative aligned perfectly with our thinking. We had high expectations, to say the least, but those expectations were exceeded beyond all our hopes. The women were incredibly dedicated. Everyone completed the program; only two missed one or two sessions here and there, but overall, everyone finished the group and made remarkable progress, transformations and reflections. They were also able to implement what they learned in practice by the next session, even though only a week had passed. It was truly fantastic.*”

(Counselor, secondary partner shelter, version 3 in a weekly format)

The final rounds of interviews, however, highlighted that PAR-inspired intervention development is, or can be, an ongoing process, in which new and improved versions spark additional ideas for improvement. One of the shelters proposed that the manual could benefit from including even more research, including research generated by the project. Strengthening the descriptions of the project’s research background was seen as particularly important, in light of the broader social policy context’s expectations and demands for evidence-based approaches.

“*If one envisages that this (referring to the intervention) is made permanent and given that women’s shelters are undergoing a process of professionalization, it is important to us to be able to refer to research. There is so little research on material specifically developed for women’s shelters. Therefore, I genuinely believe that even if it (referring to the manual) becomes somewhat heavy, the benefits will still outweigh the drawbacks.*”

(Counselor, secondary partner shelter, version 3)

Large-scale testing and collaboration with new partner shelters deepened our understanding of the intervention’s relevance and potential contribution, as well as the organizational conditions conducive to its implementation. Multi-site testing contributed strongly to the cumulative evidence supporting the view that *Strength to Move On*, version 3, has the potential to benefit victim-survivors who struggle to reorient and rebuild their lives post-separation. Prompted by the diversity of organizational conditions, implementation experiences, and the fact that future shelters would have to rely entirely on the manual, the intervention was revised one final time to better reflect the diverse circumstances of shelters and needs.

#### Key Lessons of Larger-Scale Evaluation

The fourth phase, like previous development stages, offered several opportunities to learn. The first key lesson from this multi-site round of implementation is that few shelters have experience working within a research project with a systematic evaluation protocol. Future projects that couple intervention development with systematic data collection should not only assess partners’ willingness and capacity to implement an intervention but also take time to introduce the project’s core methodological principles and basic research protocols for surveys and interviews. The second lesson for intervention developers who adopt a multi-site design with implementation cycles that vary considerably is to select a suitable project management tool early on and, as needed, modify it to enable consistent oversight and dialog with multiple test sites that have different start and end dates and different paces (in our case, weekly vs. bi-weekly group meetings).

Finally, as the compliance process continued to delay certain project activities, an emerging lesson was to continuously monitor the relevant policy field, not only at the beginning of the process. With the primary partner in a relatively stable financial position at the project’s inception, little attention was paid to the general economic circumstances of shelters during initial planning, or to the impacts of potential policy shifts. However, as the intervention development moved beyond the primary test environment and sought partnerships with previously unaffiliated shelters (phases III and IV), it became apparent that many Swedish shelters struggled to navigate a complex compliance process while also serving their community of IPV victim-survivors and engaging in PAR-inspired intervention development. Contrary to the intervention development literature, which tends to highlight policy analysis as part of preparatory work, it is advisable for future intervention developers to remain alert to changes in the policy arena and their implications. Through continuous monitoring and assessment of contextual factors that may influence partners’ resource realities and, in turn, partners’ capacity to engage in the development process and research, future developers might establish a degree of preparedness.

## 4. Overarching Transferable Lessons Learned

The preceding section outlined the four phases of intervention development and the key lessons for each phase. Some lessons learned, however, were more overarching in nature. This section synthesizes lessons learned and offers transferable advice to assist future intervention developers engaged in PAR-inspired and/or iterative design work.

### 4.1. Relationship Building and Ideological Alignment—Situating the IPV Survivor at the Center

Developing an intervention can be viewed as a series of decisions, both small and large. From the early development phases (I–II) of relationship building, collaborative intervention design, and small-scale piloting, it was clear that the primary partner’s practices strongly reflected a feminist ethics of care. The non-profit Swedish shelter system is rooted in the Swedish women’s liberation movement, embracing ideals of egalitarianism, equality, and empowerment, and it provides support in a spirit of sisterhood, with counselors approaching help-seeking IPV victim-survivors as equals [9]. Furthermore, the longstanding tradition of feminist peer support creates a unique organizational culture and set of practices. The shelter’s deep-seated modus operandi of approaching IPV holistically and placing the IPV victim-survivor and her experiences and needs at the center of each decision was adopted as a joint guiding principle. The same ideologically inspired practices were also found in different forms among secondary partners who joined later. It is highly advisable to spend sufficient time to understand each other’s worldviews, identify and explore the theoretical frameworks that guide the worldviews of collaborating partners, and prioritize alignment with them before embarking on an extended development project.

### 4.2. The Value of PAR, Epistemic Equality, and Multidisciplinary Collaboration

The project began with a clear vision of working closely with the primary partner’s management and counselors. As described earlier, drawing on PAR approaches and iterative design methods, Phases I–III were characterized by collaborative exploration and co-creation that leveraged team members’ diverse backgrounds and experiences. Guided by the PAR approach, the project drew systematically on the primary partner’s in-depth knowledge of the end-beneficiaries—IPV victim-survivors—and combined their practice-based experiential knowledge with theory and methods from psychological intervention research to inform intervention development, including the structure, modules, and individual exercises. A PAR approach advocates for epistemic equality, which in turn creates optimal conditions for dialog. The benefits of the approach became apparent at the ideation and conceptualization stage, laying the groundwork for the project team to produce a relevant prototype intervention that could be piloted. As the intervention was gradually introduced to additional shelters (Phases III and IV), the project actively sought out the new partners’ experiences to ensure the intervention was firmly grounded and informed by a multitude of perspectives. The semi-structured interviews approached all shelter counselors as experts, recognizing not only their valuable practical expertise but also their privileged position as direct observers of participants’ engagement with the intervention.

At the end of the project, it was clear that the benefits of a PAR-inspired approach emphasizing epistemic equality are manifold. First, it empowers practitioners to draw on critical insights from direct work with IPV victim-survivors and enables researchers to organize and integrate practitioners’ knowledge with theory and systematic analyses of participant data. Second, feedback from the broader group of partnering shelters during Phases III and IV indicated that their inclusion in the development process fostered a sense of co-ownership, which is likely to contribute to the intervention’s long-term survival within the Swedish shelter context. Third, a non-hierarchical approach aligns ideologically with the shelter movement. Swedish shelters, as in many places around the world [11], were built on a feminist model in which women helped other women by creating close-knit protective communities around IPV victim-survivors. The organizational culture that emerged from this legacy is often marked by wariness of hierarchical arrangements. Based on the experiences in this project, it is highly recommended to pursue non-hierarchical, multidisciplinary collaboration and to actively encourage partners to engage in development as equals and experts, sharing insights, concerns, and suggestions throughout the various phases of development. The value of non-hierarchical, multidisciplinary collaboration cannot be overstated.

While alignment on core values, priorities, and work processes could be achieved relatively easily among a core group of researchers and the partnering shelters, some researchers left due to tensions over project management or key strategic decisions. Despite this, the remaining researchers sought to recover and nurture a project culture characterized by mutual respect and epistemic equality among all parties, in which the project could leverage practitioners’ unique experiences to create not only a more effective intervention but also one that was feasible given Swedish shelters’ priorities, resources, and constraints.

### 4.3. The Importance of Formal Agreements

The research team’s configuration changed significantly over the course of the development process, resulting in significant disruption and delays. Although the core research team completed the project and successfully handed over *Strength to Move On* to the primary partner shelter, the project’s hard-earned lessons offer future intervention developers an opportunity to pre-empt similar disruptions by establishing intra-project agreement on the ownership of project-related materials and data and the handling of proprietary information and copyrights among all collaborating partners. By openly discussing views on the authorship, copyright, and rights of use of the outputs produced, as well as potential opportunities for commercialization, partners are better equipped to decide whether to pursue collaboration, apply for joint research funding, or contribute to project outputs. Addressing these issues up front is particularly important in teams where perspectives on academic hierarchy and on intellectual property rights may vary.

The issues of authorship and intellectual property rights can be organized in different ways. The core group of researchers, as joint authors, held the intervention texts and manual in shared ownership until the handover to the primary partner. The final version of the intervention, including the audio files for the meditations and the manual, was officially handed over to the primary partner under an agreement that it would be available, at no charge, on request to non-profit women’s shelters in Sweden.

Authorship on research publications was determined on a case-by-case basis using the APA’s authorship determination scorecard, and all research-related output (e.g., articles, conference presentations) was made available to the project’s partner shelters and other practitioners through open access publishing.

## 5. Discussion

The continued use of *Strength to Move On* after project completion indicates it is perceived as useful to Swedish women’s shelters, that it has reached a threshold of sustainability, and that it has the potential to be a valuable addition to shelters’ methodological toolbox. The development of the intervention thus serves as a valuable case study in intervention development, particularly in the context of community support for IPV victim-survivors.

This article provides a comprehensive overview of the multi-phase intervention development process, emphasizing key lessons learned from combining PAR with iterative design approaches. The description of the development process and key lessons learned for each of the four development phases is intentionally detailed to support future developers’ planning and implementation of intervention development in close collaboration with community stakeholders. Although the intervention development was situated in the Swedish shelter context, the lessons learned are transferable to other developers interested in combining PAR and iterative design approaches to develop interventions relevant to their contexts.

Phase-specific lessons are best unpacked alongside descriptions of the related work process and activities they involve and are explored in each development phase of the article. While the main lessons learned have been presented earlier, one topic—the iterative design approach—deserves further attention because of its importance in intervention development and its relative neglect in the intervention development literature. Using an iterative design approach inherently involves repetition: planning, defining the scope, designing a prototype, testing, analyzing feedback, making improvements, and repeating the steps until the intervention meets its targets. Combining PAR with an iterative design approach provided a structured yet flexible framework, ensuring the intervention remained centered on the experiences of users and end-beneficiaries. PAR’s focus on co-creation helped capture user needs and use them systematically to refine the intervention, resulting in a final version that is both scientifically sound and contextually relevant. The patience built into iterative testing and refinement was, however, vital to success in this particular project. Finally, an iterative design approach enabled adaptation to changing internal and external conditions and to new findings.

### 5.1. Limitations and Relevance to Future Projects

While this study offers several transferable lessons for intervention developers, it has several limitations. A descriptive case study design in which much of the case material was produced or mediated by the researchers and other project partners themselves is vulnerable to unintentional interpretive biases [32]. While interpretive biases are always present to some degree, the data were produced and analyzed by all three researchers who were involved in the project. The summative data triangulation conducted in connection with the present analyses involved all three researchers and several data sources.

Another common critique is that case studies have limited relevance beyond their immediate context. The current case material emanates from one specific context—Swedish women’s shelters and, in particular, the primary partner shelter. However, with appropriate adjustments to the intervention and work processes, the intervention was scaled up to six secondary partner shelters over the course of the project. The successful implementation at partner shelters that varied across several relevant dimensions indicates a degree of transferability of our approach to intervention development and suggests that it, along with the lessons learned, could be broadly relevant to the context of Swedish women’s shelters and potentially beyond. Looking beyond Sweden, even though women’s shelters around the world share roots in the women’s movement and offer similar services to victim-survivors, contextual conditions will undoubtedly differ from the Swedish case. This study combines analytical generalizations—the transferable lessons learned—with descriptions of the specific case context in order to allow readers to assess the study’s potential contribution and relevance to their unique contexts.

The study is also, in a sense, limited in its focus on female victim-survivors of male-perpetrated IPV. However, by adopting the study’s suggested framework for intervention development, which combines PAR and emphasizes epistemic equality with iterative design approaches, intervention teams are well-equipped to identify, analyze, and adapt future interventions to the needs of new victim groups, such as IPV victim-survivors from LGBTQ relationships and/or male victims of female-perpetrated IPV and relevant community stakeholders active in these contexts. Future research should, however, test and assess the relevance of the transferable lessons across different country contexts and with other intervention beneficiaries to validate the proposed recommendations.

### 5.2. Conclusions

The project offers a framework for intervention development in close collaboration with community stakeholders and end-beneficiaries, combining participatory action research (PAR) and iterative design approaches. By emphasizing and embracing epistemic equality between researchers and stakeholders, we were able to develop an intervention with high contextual relevance and acceptability, as well as pathways for continued dissemination and long-term implementation in the Swedish women’s shelters system. This case study presents a collaborative model and workflow for intervention development in close collaboration with community-based idea-driven non-profit actors.

## Data Availability

The data that support the findings of this study are available on request from the corresponding author. The data are not publicly available due to restrictions, e.g., they contain information that could compromise the privacy and safety of research participants.
